# Effects of Corrosion Products Deposited on 304 Stainless Steel on Reduction of Se (IV/VI) in Simulated Groundwater

**DOI:** 10.3390/ma15082705

**Published:** 2022-04-07

**Authors:** Guokai Huang, Teng Li, Xian Zhang, Lingyu Wang, Daqing Cui, Miao Yang

**Affiliations:** 1 School of Chemistry, Chemical Engineering and Life Sciences, Wuhan University of Technology, Wuhan 430070, China; guokaihuang@whut.edu.cn; 2 Department of Radiochemistry, China Institute of Atomic Energy, Beijing 102413, China; linyuwang2955@163.com (L.W.); daqing.cui@studsvik.se (D.C.); 3 Collaborative Innovation Center for Advanced Steels, Wuhan University of Science and Technology, Wuhan 430081, China; xianzhang@wust.edu.cn; 4 Department of Materials and Environmental Chemistry, Stockholm University, SE-106 91 Stockholm, Sweden

**Keywords:** 304 stainless steel, simulated groundwater, selenium, corrosion behavior, reduction rate

## Abstract

Selenium (Se) is a key mobile fission product in the geological disposal of nuclear waste. It is necessary to analyze the reductive deposition behavior of iron-based materials to Se(IV) and Se(VI) in groundwater. In the present work, the corrosion behavior of 304 stainless steel in simulated groundwater (SG) and the effects of corrosion products on the dissolution of Se were investigated by electrochemical and immersion tests. Experimental results revealed that passivation films formed on 304 stainless-steel samples were destroyed by polarization measurements, forming corrosion products consisting of Fe(II) compounds, such as Fe_3_O_4_ and FeO. Corrosion products deposited on the surface of steel samples previously treated by polarization measurements in SG + CaCl_2_/Na_2_CO_3_/Na_2_SiO_3_ solutions effectively reduced soluble Se(IV) and Se(VI) during immersion tests, depositing FeSe_2_ on sample surfaces.

## 1. Introduction

Deep geological disposal is widely accepted by major nuclear power countries in the world to isolate radionuclides permanently from the biosphere [1,2]. ^79^Se is considered a key mobile fission product for the geological disposal of nuclear waste. Selenium (Se) is a high-level radioactive waste (HLW) and exists in the form of ^79^Se (unstable isotope) with a half-life of 3.77 × 10^5^ years [3,4,5]. In the process of geological disposal of HLW, nuclides are isolated by multiple barriers; moreover, nuclides can react with corrosion products when the leakage of nuclides is caused by corrosion perforation of geological disposal containers. Therefore, the mobility of Se is a key factor for the safe disposal of HLW.

Se exists in the form of different valence compounds (−II, −I, 0, +IV, +VI) [5]. The solubility of Se is influenced by its oxidation state to some extent, and its oxidation state compounds (selenite (SeO_3_^2−^) and selenate (SeO_4_^2−^)) are highly soluble under different chemical conditions, whereas Se and its selenides have very low solubility [5,6]. It has been proven that Fe(II)-containing minerals, such as green rust [7], magnetite [8,9,10,11,12], pyrite [13,14], and troilite [14], can reduce SeO_4_^2−^ and SeO_3_^2−^, and the abiotic reduction of Fe(II)-containing minerals is very high. Se is the main reduction product of several Fe(II)-containing minerals. Iron selenide (FeSe) was found to be the main reduction product of magnetite and green rust selenite [4].

Fe(II)-containing minerals generally exist in deep geological media, and abiotic reduction has an important effect on the mobility of Se in groundwater [15]. Mingliang Kang et al. [6] investigated the interaction of aqueous Se(IV) with pyrite under O_2_-free conditions at pH ranging from 4.5 to 6.6; they found that pH and iron concentration have a significant effect on the Se(IV) reaction rate and reaction product. Mingliang Kang et al. [5] investigated the influence of pH and reaction time on the formation of FeSe_2_ by reductive precipitation of Se(IV) with nanosized pyrite–greigite; they found that FeSe_2_ was formed at pH > 6.07. Teng Li et al. [2] found that an Fe(II) compound-containing passivation film was formed on the surface of pure iron in simulated groundwater containing carbonate, silicate, and calcium ions.

Fe + 2H_2_O → Fe(OH)_2_ + H_2_. (1)

Fe^2+^ + CO_3_^2^*^−^* → FeCO_3_. (2)

2Fe^2+^ + SiO_3_^2^*^−^* + 2OH*^−^* → Fe_2_SiO_4_ + H_2_O. (3)

In order to investigate the effect of corrosion products on the migration of Se in a deep geological disposal repository after the corrosion failure of the disposal vessel, 304 stainless steel was selected as the research object to analyze the reduction adsorption and deposition behavior of Se(IV) and Se(VI) by corrosion products.

## 2. Experimental

### 2.1. Materials

Sodium chloride (NaCl; AR; Sinopharm Chemical Reagent Co. LTD.(Shanghai, China)), calcium chloride anhydrous (CaCl_2_; AR; Sinopharm Chemical Reagent Co. LTD. (Shanghai, China)), sodium hydrogen carbonate (NaHCO_3_; AR; Sinopharm Chemical Reagent Co. LTD. (Shanghai, China)), sodium carbonate (Na_2_CO_3_; AR; Sinopharm Chemical Reagent Co. LTD. (Shanghai, China)), sodium metasilicate nonahydrate (Na_2_SiO_3_·9H_2_O; AR; Sinopharm Chemical Reagent Co. LTD. (Shanghai, China)), ethanol absolute (C_2_H_6_O; AR; Sinopharm Chemical Reagent Co. LTD. (Shanghai, China)), sodium selenite (Na_2_SeO_3_; AR; Chengdu Huaxia Chemical Reagent Co. LTD. (Chengdu, China)), and sodium selenate (Na_2_SeO_4_; AR; Chengdu Huaxia Chemical Reagent Co. LTD. (Chengdu, China)) were the main chemicals of this experiment.

The chemical composition of the commercial 304 stainless steel used in this experiment is presented in Table 1. For the immersion test, the 304 stainless-steel plate was cut into samples of 20 mm × 10 mm × 2 mm size. Each sample was mechanically ground to 2000 grit by silicon carbide papers, washed with ethanol, and dried. For the electrochemical experiment, the 304 stainless-steel plate was cut into specimens of 10 mm × 10 mm × 2 mm size to prepare electrodes. These working electrodes were ground to 2000 grit, polished to a mirror surface, washed with ethanol, and dried.

### 2.2. Simulated Groundwater Solution

The simulated groundwater (SG) solutions used in this experiment contained SG (10 mM NaCl + 2 mM NaHCO_3_) and different ions (Ca^2+^, CO_3_^2−^, SiO_3_^2−^). The concentrations of Ca^2+^, CO_3_^2−^, and SiO_3_^2−^ ions were set to 0 mM, 1 mM, 5 mM, 10 mM, or 20 mM by adding CaCl_2_, Na_2_CO_3_, and Na_2_SiO_3_, respectively. The chemical compositions of the as-prepared simulated groundwater solutions are listed in Table 2.

### 2.3. Immersion Solutions

The immersion solutions used in this experiment contained SG and different valence states of Se solution (Se(IV/VI)). The concentrations of Se solution were set to 30 mg/L and 100 mg/L by adding Na_2_SeO_3_ or Na_2_SeO_4_. The chemical compositions of the as-prepared immersion solutions are listed in Table 3.

### 2.4. Electrochemical Measurements

In order to analyze the corrosion behavior of 304 stainless steel in anoxic SG, the potentiodynamic polarization curves of 304 stainless-steel samples at different ion concentrations were plotted by a Zahner electrochemical workstation (Germany) at 40 °C. An electrochemical cell consisting of three electrodes was used in this experiment; 304 stainless-steel samples acted as working electrodes, a saturated Hg/Hg_2_Cl_2_ electrode worked as the reference electrode, and a platinum foil served as the counter electrode. Each solution was purged with argon gas containing 0.03% CO_2_ for 20 min to obtain a H_2_- and O_2_-free solution. The open-circuit potential of each solution was tested for 30 min. The potentiodynamic polarization curve of each solution was tested in a potential range of −0.3 to 1.6 V relative to the open-circuit potential. The potential sweep rate was set to 0.5 mV/s.

### 2.5. Immersion Test

In order to explore the corrosion behavior of 304 stainless steel, a CHI 660E electrochemical workstation was used for pitting 304 stainless steel in SG + 20 mM CaCl_2_/Na_2_CO_3_/Na_2_SiO_3_ solutions by polarization measurements. Subsequently, the 304 stainless-steel samples treated by polarization measurements were immersed in SG solutions (Table 2) for 4 weeks. The concentrations of Se in these solutions after different immersion times were determined.

### 2.6. Surface Characterization

The concentrations of Se in immersion solutions were determined by an inductively coupled plasma optical emission spectrometer (ICP-OES; Prodigy 7/Prodigy 7, Hudson, NH, USA). The surface morphology and elemental composition of corrosion products deposited on the surface of 304 stainless-steel samples after the 4 week immersion test were analyzed by a scanning electron microscope equipped with an energy-dispersive spectrometer (SEM/EDS; Zeiss Auriga FEI Quanta FEG 250 SEM/Oxford Inca X-act 2000 EDS, Hillsboro, OR, USA). The formation mechanism of corrosion products deposited on the surface of 304 stainless-steel samples after the 4 week immersion test was analyzed by a confocal Raman microscope (CRM, Renishaw, London, UK). Corrosion products deposited on the surface of 304 stainless-steel samples after the 4 week immersion test were characterized by an X-ray photoelectron spectrometer (XPS, Kratos, Manchester, UK) equipped with a monochromated Al K-α X-ray source (HV = 1486.69 eV) at 150 W.

## 3. Result and Discussion

### 3.1. Potentiodynamic Polarization Curves

**Figure 1:**
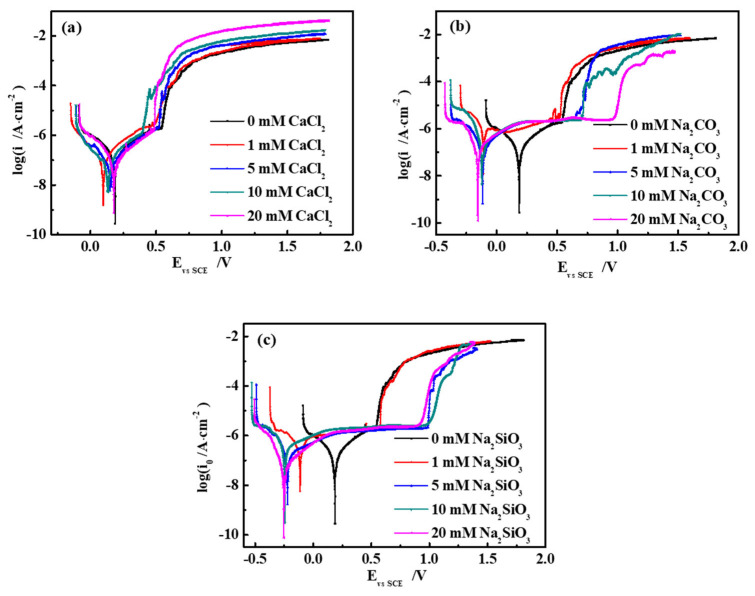
Polarization curves of 304 stainless steel in different simulated groundwater at 40 °C: (**a**) SG + CaCl_2_; (**b**) SG + Na_2_CO_3_; (**c**) SG + Na_2_SiO_3_.

The electrochemical corrosion behavior of 304 stainless steel in different SG solutions was analyzed by potentiodynamic polarization measurements at 40 °C. The polarization curves of 304 stainless steel in different SG solutions are displayed in Figure 1, and electrochemical parameters obtained from the fitted curves are listed in Table 3.

It is noticeable from Figure 1 that, in SG + CaCl_2_ solutions, the polarization curves had a similar shape, indicating that the corrosion behaviors of 304 stainless-steel samples were similar under the influence of different concentrations of CaCl_2_. The anodic polarization curve only had an activation dissolution zone. When the potential reached the pitting potential, the passivation film on the steel surface ruptured, and the corrosion current density dramatically increased. In the SG + Na_2_CO_3_/Na_2_SiO_3_ solutions, the polarization curves of 304 stainless steel had different forms. When the ion concentration was 0 mmol/L, the anodic polarization curve only had an activated dissolution zone. When the ion concentration continued to increase, the anodic polarization curve had a stable passivation zone. When the potential reached the pitting potential (Epp), the passivation film on the steel surface ruptured, and the corrosion current density dramatically increased. The Epp of 304 stainless steel was more positive as compared to the pitting potential of the steel in low-concentration solutions.

**Figure 2:**
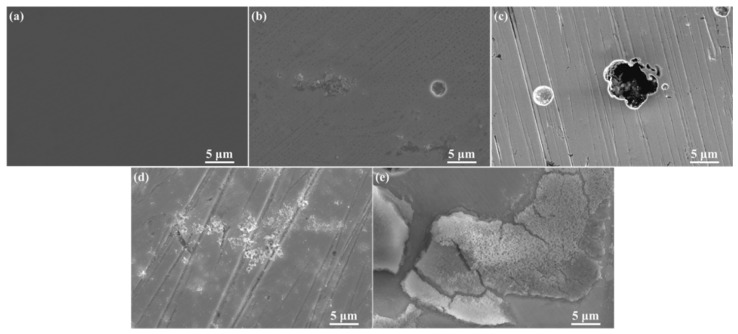
The SEM images of 304 stainless steel treated by polarization measurement in different solutions: (**a**) nontreated; (**b**) SG; (**c**) SG + CaCl_2_; (**d**) SG + Na_2_CO_3_; (**e**) SG + Na_2_SiO_3_.

It is evident from Table 4 that, in the SG solution containing CaCl_2_, the current density was 1.72 × 10^−6^ A·cm^−2^ and the Epp was around 0.5 V. In the SG solutions containing Na_2_CO_3_ and Na_2_SiO_3_, a larger passivation region led to a more positive Epp. Corrosion products only existed in pitting pits at different concentrations of CaCl_2_ (Figure 2); thus, the corrosion resistance of the steel was the same in different SG solutions. When different concentrations of Na_2_CO_3_/Na_2_SiO_3_ were added, corrosion products accumulated in a layer (Figure 2), increasing the corrosion resistance of the steel in the solutions.

### 3.2. Characterization of Corrosion Products and the Passivation Film

In order to analyze the morphology and composition of the corrosion products and passivation film, SEM/EDS measurements were performed on the surface of 304 stainless steel treated by polarization measurement in SG, SG + 20 mmol/L CaCl_2_, SG + 20 mmol/L Na_2_CO_3_, and SG + 20 mmol/L Na_2_SiO_3_ solutions.

It is clear from Figure 2 that the nontreated sample surface did not have any corrosion products and pits. In the SG solution, the presence of a small number of granular corrosion products was found near the pit. In the SG + 20 mmol/L CaCl_2_ solution, granular corrosion products only existed in pits. In the SG + 20 mmol/L Na_2_CO_3_ solution, the number of granular corrosion products increased greatly, and they existed as clusters. In the SG + 20 mmol/L Na_2_SiO_3_ solution, the number of corrosion products dramatically increased, and they existed as agglomerates, forming a thin corrosion product layer. Corrosion products generated by the reaction of CO_3_^2−^/SiO_3_^2^^−^ with the steel formed a dense passivation film on the steel surface, generating a protective effect on the steel matrix.

**Figure 3:**
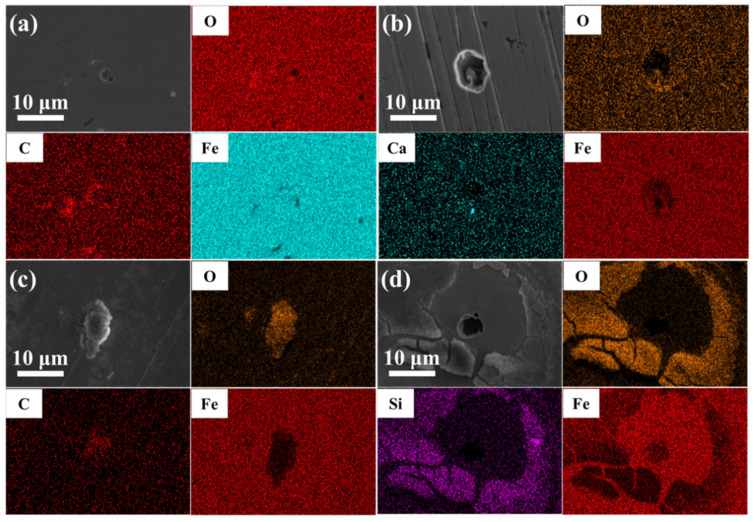
The EDS analysis of 304 stainless steel treated by polarization measurements in different solutions: (**a**) SG; (**b**) SG + CaCl_2_; (**c**) SG + Na_2_CO_3_; (**d**) SG + Na_2_SiO_3_.

**Figure 4:**
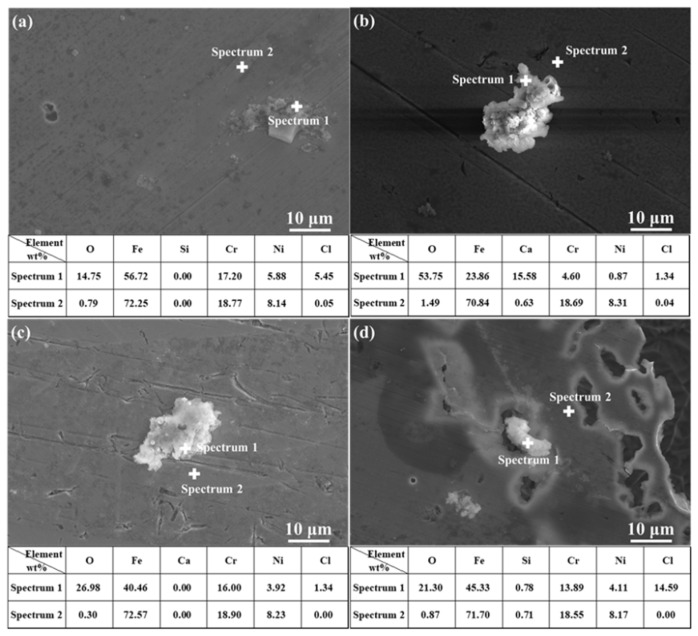
The EDS analysis of 304 stainless steel treated by polarization measurements in different solutions: (**a**) SG; (**b**) SG + CaCl_2_; (**c**) SG + Na_2_CO_3_; (**d**) SG + Na_2_SiO_3_.

It is noticeable from Figure 3 and Figure 4 that, after being treated by polarization measurements in the SG solution, corrosion products were mainly composed of Fe and O. After being treated by polarization measurements in the SG + CaCl_2_ solution, corrosion products generated on the steel surface were mainly composed of Fe, O, and Ca, and they were mainly calcium-rich products and Fe-based compounds, indicating that Ca^2+^ ions were involved in the corrosion reaction. After being treated by polarization measurements in the SG + Na_2_CO_3_/Na_2_SiO_3_ solutions, corrosion products were also Fe-based compounds. After the addition of Na_2_SiO_3_, corrosion products were mainly composed of Fe, O, and Si, indicating that SiO_3_^2−^ was involved in the corrosion reaction. Moreover, Fe-based compounds in the corrosion products mainly contained Fe(II) and Fe(III) elements, such as FeO and Fe_3_O_4_ [2].

**Figure 5:**
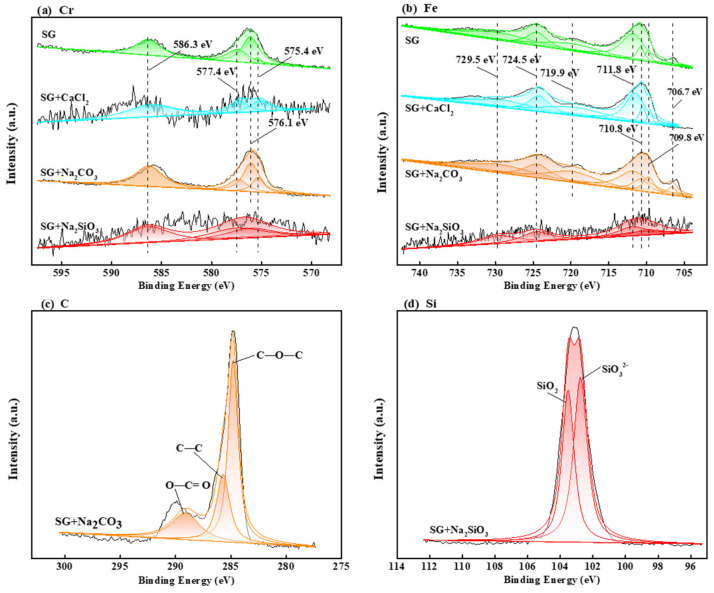
The XPS spectra of 304 stainless steel surfaces obtained after 4 week immersion in SG, SG + 20 mM CaCl_2_, SG + 20 mM Na_2_CO_3_, or SG + 20 mM Na_2_SiO_3_ solutions at 40 °C: (**a**) Cr 2*p*; (**b**) Fe 2*p*; (**c**) C 1*s*; (**d**) Si 2*p*.

Furthermore, the information of products deposited on the surface of 304 stainless steel samples immersed in SG and SG + 20 mmol/L CaCl_2_/Na_2_CO_3_/Na_2_SiO_3_ solutions was obtained by high-resolution XPS spectroscopy, as shown in Figure 5.

The Cr 2*p* spectra in different solutions contained the peaks of Cr_2_O_3_ (576.1 eV/586.3 eV), CrO_2_ (575.4 eV), and Cr(OH)_3_ (577.4 eV) [16,17,18,19] (Figure 5a). Cr in the steel matrix was oxidized to Cr(II/III) compounds and formed a passivation film on the steel surface. The Fe 2*p* spectrum consisted of seven peaks [16,17,18,19,20,21] (Figure 5b); the peaks of FeOOH (711.8 eV) and Fe_2_O_3_ (710.8 eV/724.5 eV) were the most significant, while the peaks of FeO (709.8 eV) and Fe_3_O_4_ (729.5 eV) were relatively weak. The peaks of Fe (706.7 eV/719.9 eV) were the weakest, indicating that Fe in the steel matrix was oxidized to Fe(II/III) compounds and formed a passivation film on the steel surface. In the SG + 20 mmol/L Na_2_CO_3_ solution, the C 1*s* spectra of corrosion products contained the peaks of C–C (284.4 eV), C–O–C (285.7 eV), and O–C=O (289.1 eV) [21,22] (Figure 5c), indicating the possible presence of CO_3_^2−^ ions. In the SG + 20 mmol/L Na_2_SiO_3_ solution, the Si 2*p* spectra of corrosion products contained the peaks of SiO_2_ (102.6 eV) and SiO_3_^2−^ (103.7 eV) [22,23,24] (Figure 5d), indicating that ferric carbonates and silicates were formed after the immersion test.

From the above experiments and characterization analysis, it can be seen that the Fe(II) compounds produced by the corrosion of 304 stainless steel in SG solutions containing different concentrations of CaCl_2_, Na_2_CO_3_, and Na_2_SiO_3_ were Fe_3_O_4_, FeCO_3_, FeSiO_3_, etc.

### 3.3. Analysis of the Dissolution Rate of Se after the Immersion Test

**Figure 6:**
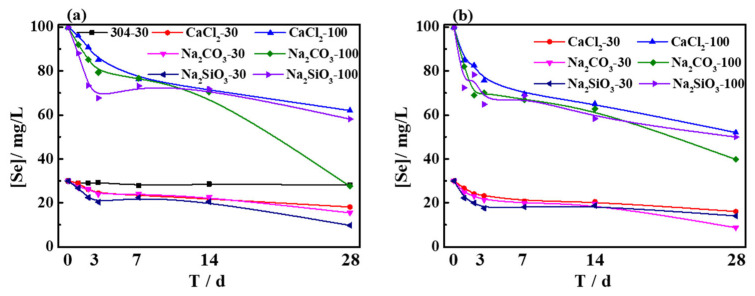
The curve of concentration of dissolved Se plotted against time after treating the 304 stainless steel by polarization measurements in different solutions: (**a**) SG + Na_2_SeO_3_; (**b**) SG + Na_2_SeO_4_.

The 304 stainless-steel samples treated by polarization measurements were directly immersed in SG + 30 mg/L Na_2_SeO_3_ solution. It is clear from Figure 6a that the concentration of soluble Se in the solution decreased slightly in the initial stage due to the adsorption effect. As the adsorption effect weakened or disappeared, the amount of dissolved Se in the solution remained negligible during the immersion period. As 304 stainless steel is a self-passivating alloy, the passivation film formed on the surface prevented the steel from reacting with Na_2_SeO_3_ in the solution; hence, only adsorption reduced the concentration of soluble Se in the solution.

Furthermore, macroscopically, no corrosion products were found on the surface of 304 stainless-steel samples treated by polarization measurements in the SG + 20 mM CaCl_2_ solution; however, a large number of corrosion products were attached to the steel surface after polarization measurements in the SG + 20 mM Na_2_CO_3_ and SG + 20 mM Na_2_SiO_3_ solutions. In addition, a large number of pits and corrosion products existed on the steel surface, as observed by SEM and shown in Figure 2, during polarization measurements. Some of the corrosion products deposited on the steel surface fell off naturally, and the loss of corrosion products in the SG solution containing Na_2_SiO_3_ was more serious. These samples were subsequently immersed in the solutions in Table 3.

It is observable from Figure 6 that, at the initial stage of immersion, the concentration of soluble Se in the solution decreased rapidly, whereas, at the later stage, the concentration of soluble Se decreased slowly. After treating the steel by polarization measurements in SG + 20 mM Na_2_SiO_3_ solution, some of the corrosion products fell off naturally, which lowered the reduction rate of soluble Se in the solution of the sample participating in the immersion test. It is clear from Table 5 that the reduction rate of soluble Se was different when the samples were treated by polarization measurements in different solutions. When the samples were treated in the SG solution containing CaCl_2_, the reduction rates of Se(IV) and Se(VI) were about 40% and 45%, respectively. When the samples were treated in the SG solution containing Na_2_CO_3_, the reduction rates of Se(IV) and Se(VI) were about 60% and 65%, respectively. When the samples were treated in the SG solution containing Na_2_SiO_3_, the values were about 50% and 50%, respectively. After polarization measurements, corrosion products were generated on the steel surface and existed in pits. Fe(II) compounds in corrosion products reduced soluble Se(VI) to soluble Se(IV) and then reacted with soluble Se(IV) through a redox reaction. As the samples were adsorbed, the concentration of soluble Se in the solution decreased rapidly in the initial immersion stage. During immersion, the adsorption process weakened, and the presence of the passivation film formed by corrosion products on the surface of the sample weakened the reaction between Fe(II) compounds and Na_2_SeO_3_, resulting in a slight decrease in the concentration of soluble Se.

The above results indicate that the samples treated by polarization measurements in different solutions led to different corrosion conditions on the surface of steel samples. In comparison to the samples not treated by polarization measurements, the treated steel samples could continuously reduce the concentration of soluble Se in the solution, indicating that the concentration of soluble Se in the solution was reduced in two ways: the adsorption process and the redox reaction between Fe(II) compounds in corrosion products and soluble Se. The number of corrosion products greatly affected the reduction of soluble Se.

### 3.4. Characterization of Corrosion Products after the Immersion Test

**Figure 7:**
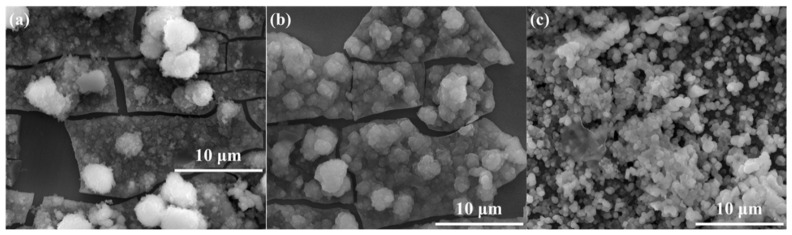
The SEM images of 304 stainless-steel samples after being treated by polarization measurements in different solutions and a 4 week immersion test in SG + Na_2_SeO_3_ solution: (**a**) SG + CaCl_2_ solution; (**b**) SG + Na_2_CO_3_ solution; (**c**) SG + Na_2_SiO_3_ solution.

The surface morphologies of corrosion products deposited on the surface of 304 stainless steel after the 4 week immersion test in SG + Na_2_SeO_3_ solutions were similar (Figure 7). Granular corrosion products formed a dense layer and existed as clusters. The corrosion products deposited on the surface of the 304 stainless-steel samples treated by polarization measurement in the SG solution containing CaCl_2_ were the lowest in number, while the corrosion products deposited on the surface of the 304 stainless-steel samples treated by polarization measurement in the SG solution containing Na_2_SiO_3_ were the greatest in number.

**Figure 8:**
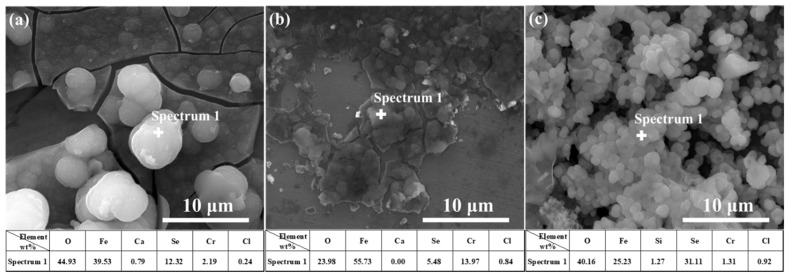
The EDS analysis of 304 stainless-steel samples after being treated by polarization measurements in different solutions and a 4 week immersion test in SG + Na_2_SeO_3_ solution: (**a**) SG + CaCl_2_ solution; (**b**) SG + Na_2_CO_3_ solution; (**c**) SG + Na_2_SiO_3_ solution.

**Figure 9:**
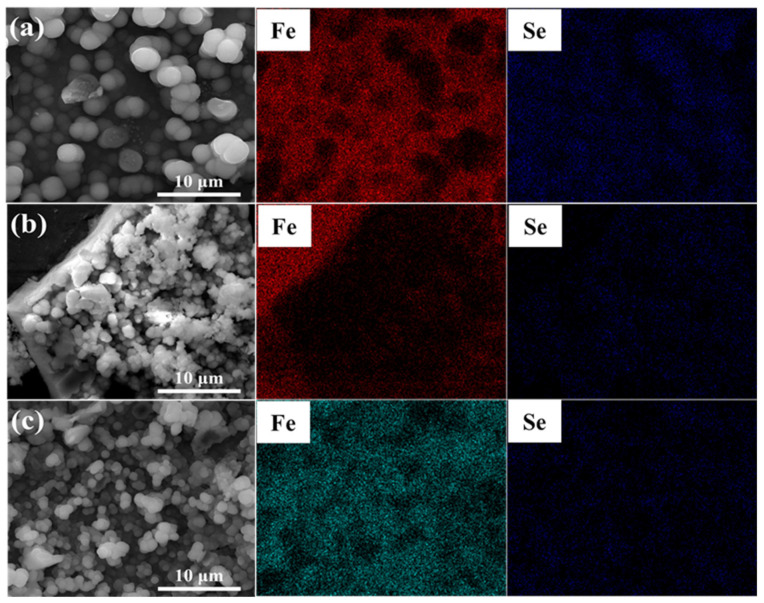
The EDS analysis of 304 stainless-steel samples after being treated by polarization measurements in different solutions and a 4 week immersion test in SG + Na_2_SeO_3_ solution: (**a**) SG + CaCl_2_ solution; (**b**) SG + Na_2_CO_3_ solution; (**c**) SG + Na_2_SiO_3_ solution.

It is noticeable from Figure 8 and Figure 9 that the main components of the corrosion products were Fe and Se, indicating that, in the immersion test, Fe(II) compounds in the corrosion products deposited on the treated steel surface were involved in the corrosion reaction. A higher number of corrosion products generated during polarization measurements indicates a greater content of corrosion products generated by Fe–Se compounds on the sample surface during the immersion test. It is preliminarily speculated that the Fe–Se compound was FeSe_2_.

**Figure 10:**
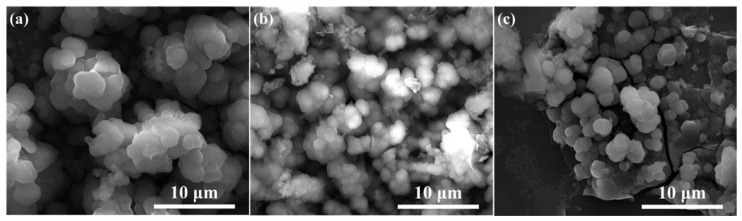
The SEM images of 304 stainless-steel samples after being treated by polarization measurement in different solutions and a 4 week immersion test in SG + Na_2_SeO_4_ solution: (**a**) SG + CaCl_2_ solution; (**b**) SG + Na_2_CO_3_ solution; (**c**) SG + Na_2_SiO_3_ solution.

**Figure 11:**
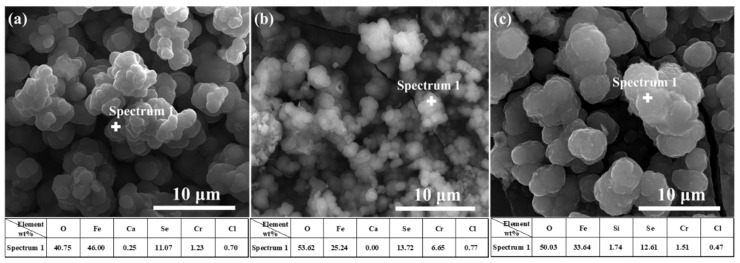
The EDS analysis of 304 stainless-steel samples after being treated by polarization measurements in different solutions and a 4 week immersion test in SG + Na_2_SeO_4_ solution: (**a**) SG + CaCl_2_ solution; (**b**) SG + Na_2_CO_3_ solution; (**c**) SG + Na_2_SiO_3_ solution.

**Figure 12:**
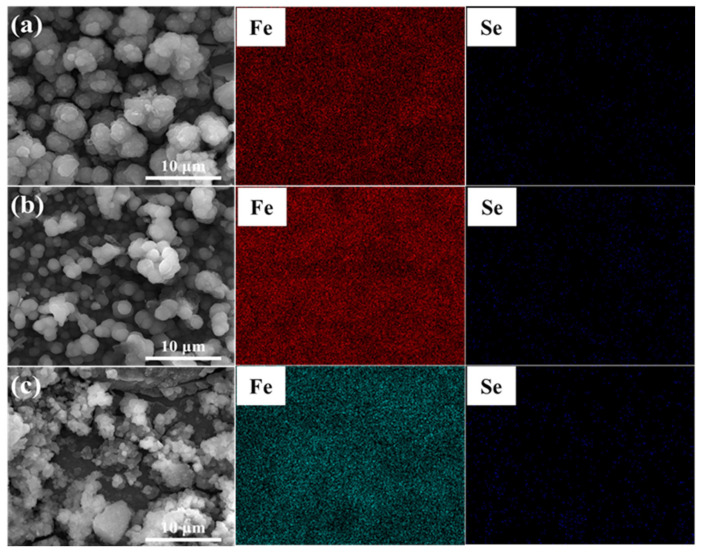
The EDS analysis of 304 stainless-steel samples after being treated by polarization measurements in different solutions and a 4 week immersion test in SG + Na_2_SeO_4_ solution: (**a**) SG + CaCl_2_ solution; (**b**) SG + Na_2_CO_3_ solution; (**c**) SG + Na_2_SiO_3_ solution.

The surface morphology of corrosion products deposited on the steel surface in the SG + Na_2_SeO_4_ solution after 4 weeks of immersion was similar to that in the SG + Na_2_SeO_3_ solution (Figure 10). Corrosion products were granular and existed as agglomerates. The main components of these corrosion products were Fe and Se (Figure 11 and Figure 12).

**Figure 13:**
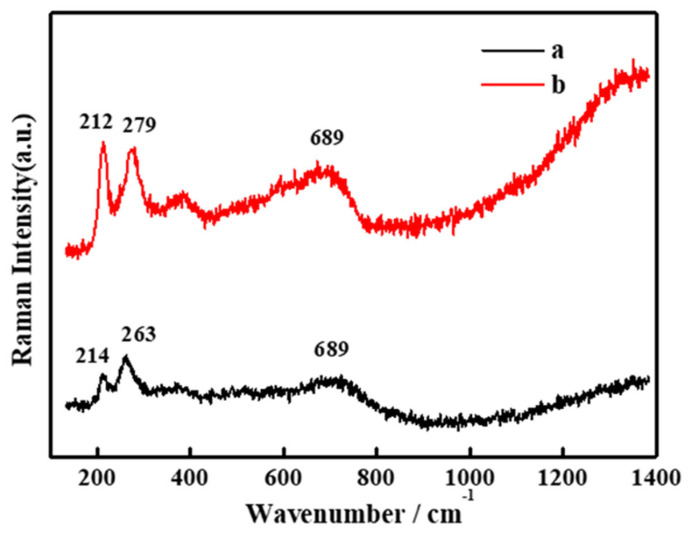
The Raman spectra of 304 stainless-steel samples after a 4 week immersion test in different solutions: (a) SG+ Na_2_SeO_3_; (b) SG+ Na_2_SeO_4_.

The Raman spectra of 304 stainless steel after the 4 week immersion test in different solutions are displayed in Figure 13. Corrosion products deposited on the steel surface were mainly composed of Fe–Cr spinel (689 cm^−1^) and FeSe_2_ (214 cm^−1^, 263 cm^−1^, 212 cm^−1^, 279 cm^−1^) [25].

The above results indicate that corrosion products containing Fe(II) compounds were generated on the 304 stainless-steel surface treated by polarization measurements in SG solutions containing CaCl_2_, Na_2_CO_3_, and Na_2_SiO_3_. Soluble Se(IV) and Se(VI) in the solutions were reduced to FeSe_2_ by corrosion products. Moreover, a greater number of corrosion products generated during polarization measurements led to a greater content of FeSe_2_ formed during the immersion test.

### 3.5. Summary

#### 3.5.1. Corrosion Behavior of 304 Stainless Steel in SG

Pits and corrosion products were generated on the surface of 304 stainless steel samples treated by polarization measurements in SG solutions containing different concentrations of CaCl_2_, Na_2_CO_3_, and Na_2_SiO_3_. In the SG solution containing CaCl_2_, granular corrosion products only existed in pits. In the SG solutions containing Na_2_CO_3_ and Na_2_SiO_3_, granular corrosion products accumulated into layers and increased the corrosion resistance of 304 stainless steel in the solutions. The main corrosion products were Fe_3_O_4_ and FeO [2,26,27].

Anodic reaction:

Fe → Fe^2+^ + 2e^−^. (4)

Cathodic reaction:

2H_2_O + 2e^−^ → 2OH^−^ + H_2_. (5)

Total reaction:

Fe + 2H_2_O → Fe(OH)_2_ + H_2_, (6)

Fe^2+^ + CO_3_^2^^−^ → FeCO_3_, (7)

2Fe^2+^ + SiO_3_^2^^−^ + 2OH^−^ → Fe_2_SiO_4_ + H_2_O. (8)

#### 3.5.2. Effects of Corrosion Products on the Dissolution of Se

Corrosion products deposited on the surface of 304 stainless-steel samples treated by polarization measurements in different SG solutions containing CaCl_2_, Na_2_CO_3_, and Na_2_SiO_3_ contained different amounts of Fe(II) compounds, which reacted with soluble Se to produce FeSe_2_ during the 4 week immersion test [6].

2HSeO_3_*^−^* + Fe^2+^ + 10H^+^ + 10e^−^ → FeSe_2_ + 6H_2_O. (9)

It was found that Fe(II) compounds could reduce soluble Se at pH = 8.2.

SeO_4_^2^*^−^* + Fe^2+^ + H^+^ → Fe^3+^ + SeO_3_^2^*^−^* + OH*^−^,* (10)

2SeO_3_^2^*^−^* + Fe^2 +^ + 6H^+^ + 10e*^−^* → FeSe_2_ + 6OH*^−^*. (11)

A greater number of corrosion products on the surface of 304 stainless steel led to a higher amount of reduced Se in the immersion solutions. The lowest number of corrosion products was deposited on the surface of 304 stainless steel treated by polarization measurements in the SG solution containing CaCl_2_, and corrosion products only existed in pitting pits. The deposition amount of corrosion products on the surface of 304 stainless steel treated by polarization measurements in the SG solutions containing Na_2_CO_3_ and Na_2_SiO_3_ increased. However, during polarization measurements, some corrosion products deposited on the steel surface fell off, and the loss of corrosion products in the SG solution containing Na_2_SiO_3_ was more serious. The reduction rate R of soluble Se was calculated as R = 1 − C_1_/C_0_, where C_0_ is the initial concentration of soluble Se, and C_1_ is the final Se concentration after the immersion test; the corresponding results are presented in Table 5.

## 4. Conclusions

1. Polarization measurements were executed on 304 stainless-steel samples in SG solutions containing CaCl_2_, Na_2_CO_3_, and Na_2_SiO_3_. Corrosion products containing Fe(II) compounds, such as FeO and Fe_3_O_4_, were generated on the surface of steel samples.
2. The reduction rate of soluble Se was different when the samples were treated by polarization measurements in different solutions. When the samples were treated in the SG solution containing CaCl_2_, the reduction rates of Se(IV) and Se(VI) were about 40% and 45%, respectively. When the samples were treated in the SG solution containing Na_2_CO_3_, the reduction rates of Se(IV) and Se(VI) were about 60% and 65%, respectively. When the samples were treated in the SG solution containing Na_2_SiO_3_, the reduction rates of Se(IV) and Se(VI) were about 50% and 50%, respectively.
3. In Se-containing solutions, Fe(II) compounds in corrosion products reduced soluble Se(VI) to soluble Se(IV), and Fe(II) compounds reacted with soluble Se(IV) through a redox reaction.
4. A large number of corrosion products were generated on the surface of 304 stainless-steel samples treated by polarization measurements. A large amount of FeSe_2_ was produced on the surface of 304 stainless-steel samples by the 4 week immersion test, and the reduction rate of soluble Se was high.

## Figures and Tables

**Table 1:** Chemical composition of the 304 stainless steel.

	Composition (wt.%)								
	C	S	P	Si	Sn	Mn	Ni	Cr	Fe
304 stainless steel	0.08	0.003	0.04	-	0.8	1.8	9	19	balance

**Table 2:** Chemical composition of simulated groundwater.

Solution	Chemicals Concentration (mM)				
	NaCl	NaHCO_3_	CaCl_2_	Na_2_CO_3_	Na_2_SiO_3_
SG	10	2	0	0	0
SG + CaCl_2_	10	2	1	0	0
	10	2	5	0	0
	10	2	10	0	0
	10	2	20	0	0
SG + Na_2_CO_3_	10	2	0	1	0
	10	2	0	5	0
	10	2	0	10	0
	10	2	0	20	0
SG + Na_2_SiO_3_	10	2	0	0	1
	10	2	0	0	5
	10	2	0	0	10
	10	2	0	0	20

**Table 3:** Chemical composition of the solution for the immersion test.

	Chemicals Concentration			
	NaCl	NaHCO_3_	Na_2_SeO_3_	Na_2_SeO_4_
SG + Na_2_SeO_3_	10 mM	2 mM	30 mg/L	0
	10 mM	2 mM	100 mg/L	0
SG + Na_2_SeO_4_	10 mM	2 mM	0	30 mg/L
	10 mM	2 mM	0	100 mg/L

**Table 4:** Electrochemical parameters obtained from the fitted curves.

40 °C		mM	0	1	5	10	20
	i/E_pp_						
CaCl_2_	i/A·cm^−2^		1.72 × 10^−6^	2.67 × 10^−6^	1.91 × 10^−6^	1.36 × 10^−6^	1.66 × 10^−6^
	Epp/V		0.545	0.498	0.524	0.400	0.487
Na_2_CO_3_	i/A·cm^−2^		1.72 × 10^−6^	3.03 × 10^−6^	2.74 × 10^−6^	2.37 × 10^−6^	2.46 × 10^−6^
	Epp/V		0.545	0.528	0.645	0.703	0.969
Na_2_SiO_3_	i/A·cm^−2^		1.72 × 10^−6^	1.98 × 10^−6^	2.11 × 10^−6^	2.67 × 10^−6^	2.77 × 10^−6^
	Epp/V		0.545	0.572	0.988	0.981	0.908

**Table 5:** The soluble Se reduction rate in the immersion test.

	Reduction Rate (%)		
	SG + 20 mM CaCl_2_	SG + 20 mM Na_2_CO_3_	SG + 20 mM Na_2_SiO_3_
SG + 30mg/L Na_2_SeO_3_	39.48	48.32	67.36
SG + 100mg/L Na_2_SeO_3_	37.98	72.46	41.86
SG + 30mg/L Na_2_SeO_4_	46.28	70.76	53.08
SG + 100mg/L Na_2_SeO_4_	47.98	60.22	50.02

## Data Availability

The study did not report any data.
